# Innovative solutions for language growth: the impact of problem-based learning via DingTalk on Chinese undergraduates’ business vocabulary amid COVID-19

**DOI:** 10.3389/fpsyg.2023.1289575

**Published:** 2023-11-16

**Authors:** Lixuan Sun, Hui Dong, Xiaotian Zhang

**Affiliations:** ^1^Department of English, College of Foreign Languages, Northeast Forestry University, Harbin, Heilongjiang, China; ^2^Department of English, School of Western Languages and Cultures, Harbin Normal University, Harbin, Heilongjiang, China

**Keywords:** business vocabulary, COVID-19, DingTalk, language acquisition, problem-based learning, remote learning

## Abstract

Amidst the COVID-19, which has necessitated the widespread use of distant learning, there has been a notable increase in the recognition and utilization of inventive pedagogical methods and technological tools in the field of language teaching. The primary objective of this research is to assess the effects of DingTalk-based PBL on the business vocabulary growth of Chinese undergraduates during the pandemic, with a particular focus on remote learning environments. This mixed-methods research employed a sample of 58 participants. The study involved both quantitative vocabulary assessments and qualitative interviews. The quantitative assessments aimed to measure the impact on vocabulary scores, while qualitative interviews were conducted to gather insights into participants’ experiences and perceptions regarding DingTalk-based PBL. The quantitative assessment revealed a significant improvement in business vocabulary scores among the participants who underwent DingTalk-based PBL. This result indicates the platform’s potential to enhance language acquisition. The qualitative interviews provided further insights, with participants expressing positive attitudes toward DingTalk-based PBL. They emphasized its capacity to sustain engagement, foster collaboration, and bridge the gap between remote learning and effective language acquisition. These findings underscore the transformative potential of DingTalk-based PBL in language education, especially in the context of challenges posed by the pandemic. While recognizing the constraints of this study, such as its limited duration and restricted contextual applicability, the research encourages further investigation into sustainable vocabulary expansion, the development of multifaceted language abilities, and the integration of these platforms into emerging hybrid educational frameworks. This study makes a valuable contribution to the ongoing discourse regarding novel technology-based methods in language instruction, providing relevant insights applicable to both present and future educational contexts.

## 1. Introduction

The current state of education has experienced significant upheavals as a result of the worldwide COVID-19 pandemic (Zemrani et al., 2021; Tilak and Kumar, 2022). Educational institutions globally have been driven to rapidly adjust their instructional methods to remote learning modes in order to assure the uninterrupted provision of education (Bozkurt et al., 2020; Leo et al., 2021). The process of transitioning to online learning has posed significant difficulties for language education, as the essential components of classroom contact, and linguistic immersion are integral to the development of language proficiency (Ali et al., 2020; Stickler et al., 2020). In the present setting, the significance of creative solutions that effectively connect conventional language training with virtual learning environments cannot be overstated. The process of acquiring language skills, particularly in specialized areas like business terminology, necessitates a thorough methodology that incorporates practical implementation, analytical reasoning, and active involvement in real-life situations (Alshare and Sewailem, 2018; Biletska et al., 2021; Ali et al., 2022). The conventional approach of memory through repetition and reliance on lectures inside the classroom setting frequently proves inadequate in cultivating these characteristics (Shapiro et al., 2017). Problem-Based Learning (PBL) is based on the underlying belief that the process of learning is most efficient when it occurs within a specific context, involves active participation, and fosters collaboration among students (Marra et al., 2014; Ali, 2019). This approach enables students to engage with authentic, real-world problems and utilize their theoretical knowledge in practical situations (Chen et al., 2021).

In the wake of the COVID-19 pandemic, educational institutions worldwide faced the formidable task of ensuring that students continue to receive quality education in a remote setting (Alasmari, 2021; Doll et al., 2021). During this crisis, digital platforms have emerged as conduits for delivering pedagogical innovation. DingTalk, a versatile online learning platform, presents an opportune solution to the challenges posed by remote education (Shu and Gu, 2023). With its comprehensive suite of features, DingTalk not only facilitates virtual classrooms but also integrates problem-based learning methodologies, thereby enabling educators to replicate real-world scenarios and enhance engagement (Taber and Li, 2021). This study aims to investigate the possible transformative effects of problem-based learning strategy through the use of DingTalk in developing the business vocabulary of Chinese undergraduate students during the COVID-19 pandemic. The primary aim of this study is to examine the influence of problem-based learning, implemented through the DingTalk framework, on the development of business vocabulary among Chinese undergraduate students and solve the following research questions.

RQ1: Is there a significant difference in the scores of business vocabulary between Chinese undergraduates who receive the DingTalk based PBL and those who do not go through this intervention during COVID-19?

RQ2: What are the attitudes of students toward the DingTalk based PBL during the COVID-19?

This investigation specifically focuses on a time period characterized by social distancing measures and the adoption of distant learning. The comprehension of the impacts of problem-based learning through the utilization of DingTalk on language development has significant importance for multiple rationales. This statement elucidates the capacity of novel pedagogical tactics to be flexibly applied in virtual environments, allowing educators to customize their approaches to align with the requirements of remote education. Furthermore, conducting a study on the effects of problem-based learning through the utilization of DingTalk, with a specific focus on business vocabulary, addresses the changing requirements of the contemporary labor force, where proficient communication within specialized fields holds great significance. This study makes a valuable contribution to the wider discourse on educational resilience and creativity in times of crisis, highlighting the transformative potential of technology in driving beneficial advancements in the field of education.

## 2. Literature review

The field of language instruction has experienced a notable shift in recent years, propelled by developments in technology, evolving pedagogical approaches, and the unanticipated shocks brought about by the COVID-19 pandemic (Bryson and Andres, 2020; Erarslan, 2021). This section explores the relevant literature, analyzing the difficulties presented by the epidemic in the context of conventional language training, the fundamental concepts that support problem-based learning (PBL), and the distinctive characteristics of DingTalk’s platform that facilitate PBL in a virtual setting.

### 2.1. Challenges in remote language education during COVID-19

The onset of the COVID-19 pandemic ushered in an era of unprecedented disruptions in education, including the field of language learning (Ubaedillah et al., 2021; Upor, 2023). Language education, traditionally known for its immersive and interactive nature, encountered formidable challenges when abruptly transplanted into remote learning environments. This section delves into the multifaceted challenges that emerged within the context of remote language education during the pandemic.

#### 2.1.1. Limited authentic interaction

One of the primary challenges of remote language education lies in the limitation of authentic interaction (Gidiotis, 2021; Vellanki et al., 2022). Language learning thrives on real-time communication, peer collaboration, and face-to-face engagement, all of which were compromised in virtual settings (Bower et al., 2017). The absence of immediate feedback, spontaneous conversation, and non-verbal cues hindered the natural language acquisition process (Moussalli and Cardoso, 2020). Students’ ability to grasp nuances of pronunciation, intonation, and cultural context, integral to effective language learning, faced hindrances in the digital realm (Marzuki and Indrawati, 2023; Sotlikova and Haerazi, 2023).

#### 2.1.2. Diminished cultural exposure

Language proficiency is closely linked to cultural exposure, enabling learners to understand context, idiomatic expressions, and cultural subtleties (Bingol, 2023). In remote learning environments, learners were deprived of the immersive cultural experiences typically encountered through physical presence in foreign language contexts (Rosenbusch et al., 2020; Wang and Fang, 2020). The inability to engage in authentic cultural activities, interact with native speakers, and experience cultural diversity directly impacted the holistic language learning experience (Wang, 2016).

#### 2.1.3. Reduced spontaneous practice

Effective language learning entails consistent and spontaneous practice (Ali et al., 2018; Bibauw et al., 2019; Saito and Plonsky, 2019). Classroom environments facilitate impromptu discussions, role-playing, and spontaneous conversations that reinforce language skills. Remote learning, on the other hand, often relegated language practice to scheduled sessions, diminishing the organic and unscripted nature of language acquisition (Kiraly and Signer, 2017). The lack of casual linguistic interaction hindered learners’ ability to internalize and apply language skills in diverse situations.

#### 2.1.4. Limited access to resources

Remote learning accentuated disparities in access to resources, affecting language learners’ access to digital tools, textbooks, and language-learning platforms (Blyth and Thoms, 2021). Learners with limited internet connectivity or inadequate technological resources faced barriers in accessing online language materials (Ezra et al., 2021). This digital divide exacerbated existing inequities in education and marginalized learners’ opportunities for effective language acquisition.

#### 2.1.5. Reduced motivation and engagement

Remote learning presented unique challenges to maintaining learner motivation and engagement (Moorhouse and Kohnke, 2021). The isolation of learners from their peers and instructors, along with the blurred boundaries between academic and personal spaces, contributed to reduced motivation and a sense of detachment (Kim and Asbury, 2020). Learners’ commitment to language learning was tested, as the absence of immediate social rewards and accountability mechanisms led to fluctuating engagement levels.

### 2.2. Problem-based learning principles

Amidst the challenges posed by remote language education during the COVID-19 pandemic, educators and researchers have turned to innovative pedagogical approaches to mitigate the limitations of traditional instructional methods (Hazaea et al., 2021; Turnbull et al., 2021). Problem-based learning (PBL) has emerged as a transformative framework that addresses these challenges by fostering active engagement, critical thinking, and contextualized learning experiences (Samson, 2015; Gewurtz et al., 2016). This section delves into the principles that underpin PBL and its applications within language education.

#### 2.2.1. Foundations of problem-based learning

Problem-based learning is rooted in constructivist learning theories that emphasize learners’ active role in constructing knowledge (Kemp, 2011; Marra et al., 2014). It seeks to bridge the gap between theoretical knowledge and real-world application by presenting learners with authentic, complex problems that reflect challenges encountered in professional settings (Edens, 2000; Dunlap, 2005). Learners engage in self-directed exploration, hypothesis testing, and collaborative problem-solving, thereby cultivating deeper understanding and long-term retention (Hmelo-Silver, 2004).

#### 2.2.2. Core elements of problem-based learning

Problem-based learning is characterized by several core elements that contribute to its efficacy in language education: (1) Complex, Real-World Problems: PBL scenarios are designed to mimic authentic challenges faced in real-life contexts (Kiili, 2007). In the realm of language education, these problems might revolve around communication breakdowns, intercultural misunderstandings, or business correspondence (O’Dowd, 2012). (2) Active Exploration: Learners are encouraged to actively seek solutions through research, critical analysis, and collaborative dialog. This approach contrasts with traditional instruction where information is typically presented passively. (3) Self-Directed Learning: PBL places learners in the driver’s seat of their education, requiring them to identify knowledge gaps, set learning goals, and navigate the learning process independently (Barber and King, 2016). (4) Collaboration: Collaboration is intrinsic to PBL, fostering communication skills, shared problem-solving, and exposure to diverse perspectives (Karantzas et al., 2013; Almazroui, 2023).

### 2.3. Applications of problem-based learning in language education

The adaptability of PBL has led to its successful integration into language education, yielding several notable benefits: (1) Contextualized Learning: PBL enables learners to apply language skills in authentic contexts, bridging the gap between theory and practice. Learners engage with language as a tool for problem-solving rather than as an isolated skill (Amerstorfer and Freiin von Münster-Kistner, 2021). (2) Critical Thinking and Problem-Solving: PBL cultivates learners’ ability to analyze, synthesize, and evaluate information critically (Puspitasari, 2020; Bano et al., 2022). This skillset is invaluable for navigating complex linguistic situations, such as negotiating meaning or interpreting idiomatic expressions. (3) Autonomy and Motivation: PBL empowers learners by giving them ownership of their learning journey. This autonomy can foster intrinsic motivation and a sense of accomplishment. (4) Active Engagement: PBL’s interactive and collaborative nature maintains learners’ engagement and minimizes the detachment often associated with remote learning environments (Foo et al., 2021).

### 2.4. DingTalk’s platform for problem-based learning

As remote language education endeavors to adapt to the challenges posed by the COVID-19 pandemic, innovative solutions are imperative (Sunita, 2020; Alcalde and García, 2021; Mavridi, 2022). DingTalk, a versatile online learning platform developed by Alibaba Group, emerges as a potent contender, integrating problem-based learning (PBL) principles within a virtual environment. This section explores how DingTalk’s platform aligns with PBL principles, fostering dynamic problem-solving and interactive language learning experiences.

#### 2.4.1. Virtual environment for collaborative learning

DingTalk’s platform provides educators with a multifunctional virtual classroom that supports synchronous and asynchronous communication (Valentina et al., 2022; Jiang et al., 2023). Through features like real-time messaging, discussion boards, and document sharing, DingTalk enables students to engage in collaborative activities irrespective of physical distance (Irawan et al., 2023). This collaborative element resonates with PBL’s emphasis on peer interaction and collective exploration. PBL thrives on student collaboration and active discussion. DingTalk offers group chat functionalities, discussion boards, and video conferencing options, allowing students to work together on the assigned problem, share their insights, and engage in meaningful discussions. The platform facilitates communication and interaction among students, replicating the collaborative aspect of traditional PBL.

#### 2.4.2. Designing authentic scenarios

Central to PBL is the presentation of authentic, real-world problems that challenge learners to apply their knowledge in practical contexts (Ali, 2019). DingTalk’s platform empowers educators to design problem scenarios that mirror industry challenges, simulating linguistic situations encountered in professional settings (Gui et al., 2021). Learners engage in language use as a tool for addressing challenges, bridging the gap between theoretical language skills and their real-world application (Weng, 2023). In a traditional PBL approach, instructors present real-world problems or scenarios to students. DingTalk enables instructors to create and share these scenarios with students through its messaging and content sharing features. Instructors can upload documents, images, or multimedia resources, providing students with the necessary materials to begin their problem-solving process.

#### 2.4.3. Interactive multimedia integration

Effective PBL necessitates the integration of diverse multimedia resources that facilitate exploration and comprehension of complex issues (Azer et al., 2013). DingTalk’s platform supports multimedia integration, enabling educators to incorporate audio, video, and interactive materials that enhance learners’ understanding of language nuances and cultural contexts (Huynh and Tran, 2023). This multimedia-rich environment encourages multisensory engagement, a crucial facet of immersive language learning (Vu et al., 2022). DingTalk’s platform offers the capability to seamlessly integrate interactive multimedia elements into the learning process. In the context of PBL, instructors can use DingTalk to present problems in multimedia formats, such as videos, interactive simulations, or multimedia case studies. This immersive content not only engages students but also enhances their understanding of the problem at hand. For instance, a business case study with interactive elements can be shared through DingTalk, allowing students to explore the details of the scenario.

#### 2.4.4. Facilitating self-directed learning

Problem-based learning encourages learners to take ownership of their education through self-directed learning (Leary et al., 2019). DingTalk’s platform supports this endeavor by enabling learners to access resources, navigate learning materials, and engage in independent research. Learners can explore language-related topics and seek solutions to linguistic challenges, mirroring the autonomous learning central to PBL. PBL encourages students to take ownership of their learning, and DingTalk supports this aspect by enabling self-directed learning. Instructors can share problem scenarios, learning resources, and guidelines on DingTalk. Students, in turn, can access these materials at their convenience, review them, and decide how they want to approach the problem-solving process. DingTalk’s user-friendly interface and organization of content make it easier for students to navigate and explore resources independently.

#### 2.4.5. Collaboration and peer learning

Collaboration is a cornerstone of PBL, promoting interaction, diverse perspectives, and shared problem-solving (Davidson and Major, 2014). DingTalk’s platform facilitates group projects, peer reviews, and collaborative discussions, enabling learners to collectively tackle linguistic challenges (Huang et al., 2020). Through peer learning, students refine their language skills, exchange insights, and develop a deeper understanding of language nuances. Collaboration is a core component of PBL, and DingTalk provides a collaborative online environment. Students can form groups or teams within DingTalk to work on problems collectively. They can share their ideas, insights, and findings through group chats and discussions, fostering peer learning. DingTalk also allows for real-time collaboration through video conferencing and screen sharing, which is particularly beneficial when students need to brainstorm solutions or present their findings to their peers.

In these ways, DingTalk serves as a platform that effectively connects PBL principles with technology. It allows for the integration of interactive multimedia content to enrich problem scenarios, supports self-directed learning by providing easy access to resources, and fosters collaboration and peer learning among students. By combining the strengths of PBL with the features of DingTalk, educators can create engaging and effective learning experiences that promote critical thinking and problem-solving skills in a remote or online educational setting.

### 2.5. Previous studies and research

The integration of problem-based learning (PBL) within virtual learning environments has garnered attention from scholars and researchers seeking innovative solutions to address the challenges posed by remote language education during the COVID-19 pandemic. This section delves into previous studies and research that have explored the effectiveness of PBL and DingTalk platform in enhancing language acquisition, particularly within the realm of vocabulary.

#### 2.5.1. PBL in vocabulary learning

In the field of vocabulary learning, several recent studies have explored the effectiveness of Problem-Based Learning (PBL) and its variants. These studies shed light on how PBL can enhance vocabulary acquisition and application, particularly in various educational and practical contexts. Lin (2015) investigated the application of PBL in an elementary school in Taiwan. The study revealed that the PBL group, engaged in learner-centered activities, outperformed the control group, especially in applying vocabulary in writing, notably at the Off-List level. Additionally, the PBL group demonstrated a better grasp of advanced vocabulary and wrote longer essays. These findings suggest that PBL enhances vocabulary application and conversation practice in elementary school language education. Mohammadi (2017) conducted a study with 64 Iranian English as a Foreign Language (EFL) learners. This study focused on the use of problem-based tasks to engage cognitive and metacognitive skills in real-life vocabulary challenges. The experimental group, selected based on Nelson vocabulary test scores, outperformed the control group in vocabulary recall and retention tests, even after a 2-week interval. These results emphasize the effectiveness of authentic problem-based learning for vocabulary acquisition. Chen et al. (2021) explored the integration of virtual reality (VR) technology into PBL for vocabulary acquisition among engineering majors. The experimental group, which used VR technology to engage with PBL scenarios and create VR videos for problem-solving, significantly outperformed the control group, leading to higher motivation for career-related English learning. This highlights the potential of VR-assisted PBL in enhancing vocabulary acquisition and motivation, particularly in career-oriented contexts. Collectively, these studies underscore the positive impact of PBL and related approaches on vocabulary learning. They emphasize the need for further research to explore the specific mechanisms and contexts through which PBL can be most effective, indicating a promising avenue for future investigations in vocabulary acquisition.

#### 2.5.2. DingTalk platform in language learning

Recent research has investigated the application of DingTalk, an advanced educational platform, in language learning, offering insights into its impact on various aspects of education. Zhou et al. (2022) examined the use of DingTalk for enhancing Chinese vocabulary acquisition through seamless learning. Their study revealed a positive impact on vocabulary development, emphasizing the potential of seamless learning for language learners. Huang et al. (2021) focused on DingTalk-based collaborative prewriting in the context of junior high school EFL learners. The findings indicated that this approach improved writing performance and reduced writing anxiety, highlighting its benefits for language learners. Yan et al. (2022) explored the motivation of Chinese English majors in oral English learning, considering the influence of WeChat and DingTalk within the framework of Self-Determination Theory. The study emphasized the importance of identified and integrated regulation for sustained motivation, particularly in the context of projects with higher student responsibility. Generally, these studies showcase the growing role of technology platforms like DingTalk in language education and its potential to impact vocabulary acquisition, writing performance, and learner motivation. They collectively point to the need for further exploration in this field, particularly in understanding the specific mechanisms through which these platforms can enhance language learning.

The literature underscores the urgency for innovative solutions in language education, particularly in remote settings precipitated by the COVID-19 pandemic. Problem-based learning, rooted in constructivist theories, has emerged as a viable approach to address these challenges by fostering contextual, active, and collaborative learning experiences. DingTalk’s platform, with its integrated PBL features, offers an exciting avenue for educators to replicate real-world language contexts in virtual classrooms. Building on the insights from previous studies, this paper seeks to contribute to the discourse by investigating the impact of PBL via DingTalk on Chinese undergraduates’ business vocabulary growth during the COVID-19 era.

## 3. Materials and methods

The research methodology employed in this study utilizes a sequential explanatory mixed-methods design, inspired by the work of scholars such as Creswell and Clark (2011). This approach involves an initial quantitative data collection and analysis phase, focusing on Chinese undergraduates’ business vocabulary scores. Subsequently, in the following phase, we gather qualitative data to gain insights into their attitudes toward DingTalk-based PBL. The choice of a mixed-method approach is grounded in its capacity to provide a more comprehensive understanding of the research questions, as well as its ability to offer a well-rounded perspective on the phenomena under investigation (Creswell and Clark, 2011).

### 3.1. Sequential explanatory mixed-methods research design

**FIGURE 1:**
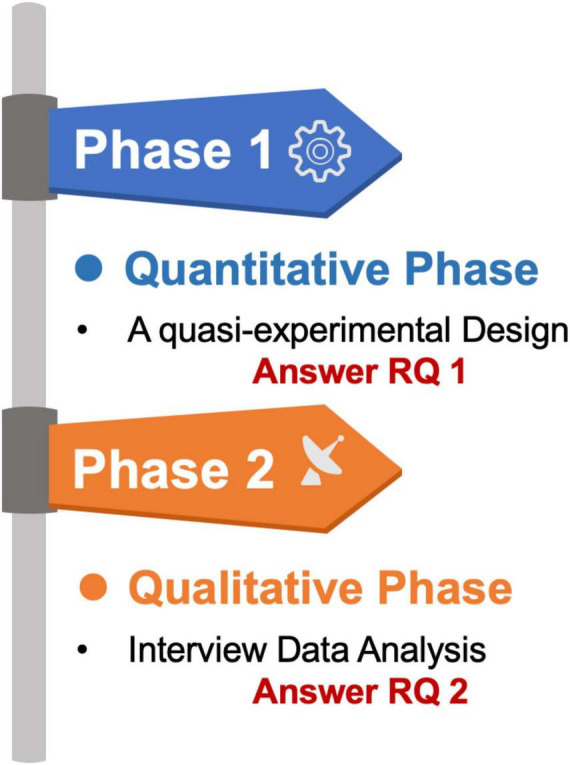
Sequential explanatory mixed-method research design.

A mixed-methods research design is employed to provide a comprehensive understanding of the impact of DingTalk-based PBL. The research design encompasses both quantitative and qualitative data collection and analysis. The quantitative component focuses on assessing the differences in business vocabulary scores between students who underwent DingTalk-based PBL and those who did not. The qualitative component involves exploring students’ attitudes toward DingTalk-based PBL. A diagram of the selected sequential explanatory mixed-method research design used in the study (Figure 1) is provided below.

### 3.2. Quasi-experimental research setting and participants

Our research was conducted at the esteemed Faculty of Foreign Languages in Harbin City, known for its excellence in language course coordination. The study took place during the challenging COVID-19 pandemic, necessitating the integration of an innovative online learning platform into daily instruction. Business vocabulary instruction became closely intertwined with the utilization of this advanced online network platform as language education evolved, making this location an ideal choice for our study. We recruited a cohort of 58 Chinese undergraduate students, each of whom met rigorous criteria for participation. To provide more clarity regarding our participant selection, these criteria were based on academic performance and course enrolment, ensuring a well-qualified and contextually relevant sample. Our sample size determination was based on a meticulous power analysis, taking into account the recommendations of Williams (2007), who advised that approximately 15 individuals per group are needed for quasi-experimental investigations. The study was facilitated by two experienced instructors with a high level of expertise in the field of business English vocabulary. The control group was directed by a single instructor who implemented a traditional business vocabulary teaching approach, while the experimental group was led by a different instructor who employed PBL and incorporated the use of DingTalk. In terms of ethical considerations, the study adhered to established guidelines and received the necessary approvals. Informed consent was obtained from all participants, ensuring their willingness to engage in the study. We maintained strict confidentiality of all participant information, and their rights were upheld throughout the research process. This ethical framework underscores the reliability and integrity of our study.

To ensure the fairness and reliability of the evaluation procedure in the study, the business vocabulary assessments, including both the pre-test and post-test, were subjected to meticulous grading by two expert raters. In this study, we employed a quasi-experimental design, with participants intentionally assigned to distinct experimental and control groups. The experimental group received PBL with DingTalk, while the control group received traditional instruction. This design allowed us to assess the impact of the intervention on vocabulary acquisition and attitudes.

### 3.3. Instrumentation

#### 3.3.1. Business vocabulary assessment (pre-test and post-test)

The statistical software SPSS 27.0 was employed to conduct a quantitative analysis on the outcomes of the business vocabulary test. The two instructors allocated students to participate in a business vocabulary test in both the experimental and control groups as a pre-test. Both groups of students are required to undertake a business vocabulary assessment, which necessitates completion within a time frame of 30 min. At the conclusion of a 12-week study, participants in both the experimental group, who were instructed using the DingTalk based PBL teaching method, and the control group, who were taught using the traditional teaching approach, were administered a business vocabulary post-test. The test was to be completed within a 30-min time frame. The reliability of the Business Vocabulary Assessment was measured with the help of the Kuder-Richardson Formula 20 (KR20), and the reliability coefficients was found to be 0.91. KR-20 values of 0.8 or higher are considered good reliability (Salkind, 2010). The test–retest reliability measures which was 0.94 show that the scales can be said to have acceptable internal consistency.

#### 3.3.2. Semi-structured interview

To gather additional insights into the students’ attitudes and impressions of the DingTalk based PBL teaching method during the COVID-19 term, interviews were conducted with the students. The interviews were conducted exclusively with students from the experimental group, as they had extensive exposure to the DingTalk based PBL teaching method for a duration of 11 weeks.

##### 3.3.2.1. Interview process transparency and reproducibility

To enhance transparency, we meticulously planned and executed our semi-structured interviews. The interview guide, developed to ensure content validity, underwent rigorous scrutiny and refinement. A panel of experts in the field reviewed and validated the questions to ensure that they captured relevant information accurately. The interview guide, was structured around key aspects of the DingTalk-based PBL teaching method, such as collaboration, engagement, and adaptability. The interviews took place in a controlled setting, ensuring consistency and reproducibility. All interviews were conducted by a single experienced interviewer who received comprehensive training in interview techniques to minimize bias and maintain consistency. This training involved mock interviews and pilot testing to fine-tune the process. Additionally, detailed notes were taken during each interview, and the recordings were transcribed verbatim, contributing to the transparency of our data collection process.

##### 3.3.2.2. Development and content validity of the interview guide

The interview guide was developed through an iterative process, involving subject matter experts and educational researchers. It was designed to cover a range of topics related to the DingTalk-based PBL teaching method. This iterative development process aimed to ensure that the questions were relevant, comprehensive, and capable of eliciting meaningful responses from participants. The setting for interviews was a designated quiet room in the educational institution, fostering a comfortable and confidential environment for participants to share their views. The interviews were conducted in one-on-one sessions and lasted approximately 30–40 min, with participants from the experimental group. The questions posed during these interviews were specifically tailored to gauge students’ experiences with the DingTalk-based PBL teaching method. Examples of questions include, “How did DingTalk facilitate your group collaboration during the 11-week period?” and “What challenges did you encounter when adapting to this teaching method?”

##### 3.3.2.3. Use of different instructors for control and experimental groups

We acknowledge that the use of two different instructors for the control and experimental groups requires clarification. This approach was taken to minimize instructor-specific bias and to isolate the effects of the teaching method itself. Both instructors were experienced and held equivalent qualifications, and they received training to ensure consistent implementation of the curriculum. Any variations in the teaching process were meticulously documented and analyzed to determine their impact on the study outcomes. This methodological decision aimed to enhance the internal validity of our study by reducing potential confounding variables related to instructor differences.

The justification for the random selection of four students from the experimental group subsequent to their involvement in the PBL + DingTalk instructional approach is outlined as follows:

Student A was selected from a pool of individuals who obtained scores ranging from 50 to 60. Student B was chosen from a group of individuals who achieved scores ranging from 60 to 70. Student C was selected from a cohort of individuals who obtained scores ranging from 70 to 80. Lastly, student D was specifically chosen from a subset of individuals who achieved scores ranging from 80 to 100. The selection of students was conducted through a rigorous evaluation process, taking into account their performance in the preceding Business English course. This approach aimed to ensure that a diverse representation of students from the class was included. Based on the university’s standards, scores falling within the range of 50 to 70 were classified as low scores. Consequently, students A and B exhibited the attributes associated with low-scoring students, whereas students C and D had qualities indicative of proficient students. The interviews were conducted in Chinese and taped to ensure comprehensive participation of the participants. Subsequently, an analysis was conducted on the responses provided by both pupils and the teacher, which were subsequently translated into English.

### 3.4. Data analysis

The quantitative portion of the study involved analyzing the pre-test and post-test scores of business vocabulary using appropriate statistical methods in SPSS 27.0. The analysis was conducted rigorously. The objective of the inquiry was to determine if there were any significant enhancements in language learning outcomes observed in the experimental group as compared to the control group. Transitioning to the qualitative phase, a rigorous methodology of thematic analysis was utilized to thoroughly examine the interview material collected from the participants. The transcriptions of the interviews were carefully examined in order to uncover recurring themes that provide insight into the attitudes and experiences of the participants regarding the novel teaching approach known as DingTalk based PBL teaching method. The integration of quantitative data analysis and qualitative insights derived from interviews yielded significant empirical evidence and enhanced the overall research findings.

## 4. Results and discussion

The findings of this study reveal the transformative potential of DingTalk-based PBL in enhancing business vocabulary among Chinese undergraduates during the COVID-19 period.

### 4.1. Results of research question 1

The business vocabulary of Chinese students was assessed by two raters in both the pre-test and post-test. The mean scores supplied by the two raters were compared between the experimental and control groups. The cumulative score amounts to a total of 20 points. In our study, we assessed the normality of the data using the Kolmogorov–Smirnov (K–S) test. The K–S test is a widely used method for determining whether a dataset follows a normal distribution. We found that the data collected for both the control group and the experimental group did not significantly deviate from a normal distribution based on the results of the K–S test. This indicates that the assumption of normality was met, allowing us to apply parametric statistical tests for data analysis. The normality of the data is a critical assumption for many statistical analyses, and by confirming its presence through the K–S test, we ensured the reliability of our statistical findings.

#### 4.1.1. Comparison of business vocabulary pre-test scores between experimental and control groups

**FIGURE 2:**
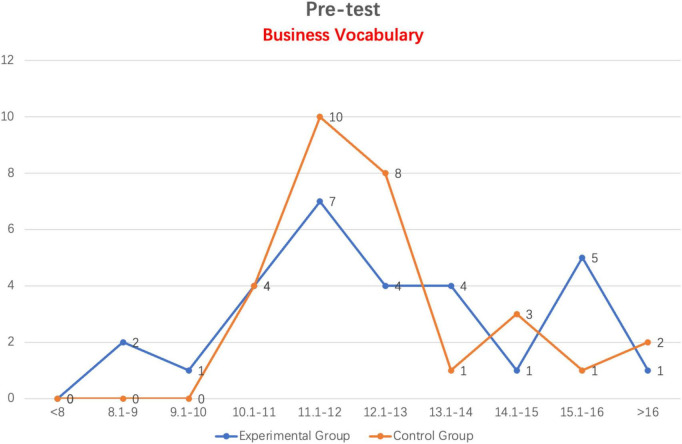
Line distribution of experimental and control groups in business vocabulary pre-test scores.

As demonstrated in Figure 2, the average scores of students in both the experimental and control groups exceed 8 points. There are two students in the experimental group with scores between 8 and 9 points. The control group’s lowest score falls between 10 and 11 points. Most students in the experimental group and the control group have scores between 11 and 12 points, and the total number of students in the control group is 10, which exceeds the number of students in the experimental group which is seven. Eight students in the control group scored between 12 and 13 points, which was double the number of students in the experimental group. In the range of 13 to 14 points, the number of students in the control group dropped to only one student, while the number of students in the experimental group remained consistent at four. Six students from the control group and seven students from the experimental group fall within the range of scores greater than 14 points.

The independent sample *t*-test in SPSS 27.0 was implemented to compare and analyze the Business Vocabulary scores of the experimental group and the control group in order to determine whether there was a significant difference between the two groups, allowing for further analysis of the pre-test data.

The Business Vocabulary pre-test scores for the experimental and control groups are provided in Table 1. The average Business Vocabulary score for 29 students in the experimental group was 12.72, while the average score for 29 students in the control group was 12.90. The control group performed marginally better than the experimental group on the Business Vocabulary test by a difference of 0.18 points. On the Business Vocabulary test, the experimental and control groups’ outcomes were comparable. Table 2 below describing *t*-test for independent samples can be employed to determine whether there is a statistically significant difference between the Business Vocabulary pre-test scores of the experimental and control groups.

**TABLE 1:** Group statistics of experimental and control groups in business vocabulary pre-test scores.

	Class	*N*	Mean	Std. Deviation	Std. Error mean
Pre-test	EG	29	12.72	2.19	0.41
	CG	29	12.90	1.76	0.33

**TABLE 2:** Independent samples test of experimental and control groups in vocabulary and word choice pre-test scores.

		Levene’s variance equivalence test		Mean equivalence *t*-test						
		**F**	**Sig.**	***t***	**df**	**Sig. (2-tailed)**	**Mean difference**	**Std. error difference**	**95% confidence interval of the difference**	
									**Lower**	**Upper**
Pre-test	Assuming equal variance	2.03	0.16	−0.33	56	0.74	−0.17	0.52	−1.22	0.87
	Assuming unequal variances			−0.33	53.52	0.74	−0.17	0.52	−1.22	0.87

Table 2 shows the results of independent sample testing for Business Vocabulary scores for the experimental and control groups. The results of the Levene’s variance equivalence test and the likelihood that the two variables are statistically significant are shown in Table 2 (Sig.). The difference in pre-test variance between the experimental group and the control group was equal, as indicated by the value of 0.16. The results (Sig 2-tailed) demonstrate that there is no significant difference in Business Vocabulary pre-test scores between the experimental and control groups since the probability of significance for the pre-test is 0.74, which is greater than the significance level of 0.05.

#### 4.1.2. Comparison of business vocabulary post-test scores between experimental and control groups

**FIGURE 3:**
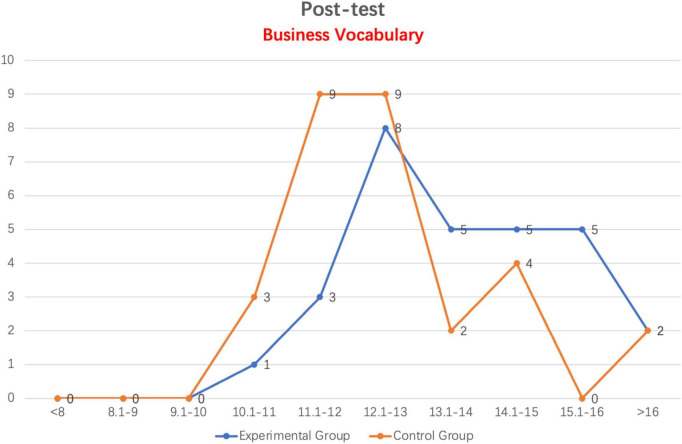
Line distribution of experimental and control groups in business vocabulary post-test scores.

As shown in Figure 3, the Business Vocabulary post-test scores of the experimental group and the control group differ significantly in the range of more than 13 points. The experimental group consists of 17 students, whereas the control group consists of just eight students. A majority of students in the control group had Business Vocabulary scores between 11 and 13 points (18 students). Simultaneously, the Business Vocabulary post-test scores of two groups of students are all greater than 10 points. In the range of scores of more than 16 points, the number of students in both groups is the same (two students).

In order to compare and analyze the Business Vocabulary scores of the experimental group and the control group, the independent sample *t*-test in SPSS 27.0 was utilized to ascertain whether there was a significant difference between the two groups, allowing for further analysis of the post-test results.

Table 3 describes the Business Vocabulary post-test scores of the experimental and control groups. The experimental group’s average Business Vocabulary post-test score was 13.98, while the control group’s score was 12.95. The experimental group’s average score on Business Vocabulary is 1.03 points higher than that of the control group. This finding implies that the experimental and control groups had distinct Business Vocabulary scores. Using the independent sample *t*-test in Table 4, it is feasible to assess whether there is a statistically significant difference between the Business Vocabulary post-test scores of the experimental and control groups.

**TABLE 3:** Group statistics of experimental and control groups in business vocabulary post-test scores.

	Class	*N*	Mean	Std. Deviation	Std. Error mean
Post-test	EG	29	13.98	1.56	0.29
	CG	29	12.95	1.68	0.31

**TABLE 4:** Independent samples test of experimental and control groups in business vocabulary post-test scores.

		Levene’s variance equivalence test		Mean equivalence *t*-test						
		**F**	**Sig.**	***t***	**df**	**Sig. (2-tailed)**	**Mean difference**	**Std. error difference**	**95% confidence interval of the difference**	
									**Lower**	**Upper**
Post-test	Assuming equal variance	0.03	0.85	2.43	56	0.02	1.03	0.43	0.18	1.89
	Assuming unequal variances			2.43	55.72	0.02	1.03	0.43	0.18	1.89

Table 4 shows the results of independent sample testing of Business Vocabulary scores for both the experimental and control groups. The significance of the two variables is indicated by the results of Levene’s variance equivalence test in Table 4 (Sig.). The post-test variances of the experimental and control groups are equivalent, as indicated by the value 0.85. The data (Sig 2-tailed) reveal a statistically significant difference in Business Vocabulary post-test scores between the experimental and control groups, with a post-test probability of significance (0.02) less than the significance level of 0.05.

#### 4.1.3. Comparison of business vocabulary pre-test and post-test scores in the experimental group

**FIGURE 4:**
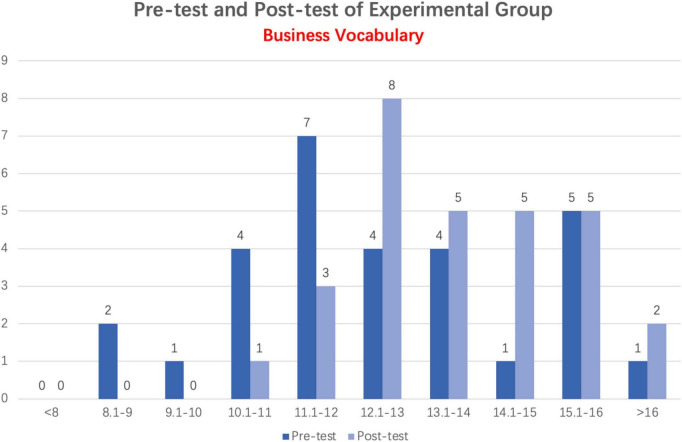
Column distribution of business vocabulary pre-test and post-test scores in experimental group.

As illustrated in Figure 4, the experimental group’s Business Vocabulary post-test scores are significantly higher than their Business Vocabulary pre-test scores. The post-test scores of most students exceeded 12 points, reaching 25. A total of 15 students scored more than 12 points on the pre-test, whereas majority of students scored fewer than 12 points.

To evaluate whether there has been a relative improvement, the Business Vocabulary pre-test and post-test scores of the experimental group are compared employing a paired sample *t*-test. The results of the experiment are displayed in Tables 5, 6.

**TABLE 5:** Paired sample statistics of business vocabulary pre-test and post-test scores in the experimental group.

		Mean	*N*	SD	Std. Mean
Pair 1	Pre-test score	12.72	29	2.19	0.40
	Post-test score	13.76	29	1.66	0.31

**TABLE 6:** Paired sample test of business vocabulary pre-test and post-test scores in the experimental group.

		Paired difference							
		**Mean**	**SD**	**Std. Mean**	**95% confidence interval of difference**		***T***	**df**	**Sig.**
					**Lower limits**	**Upper limits**			
Pair 1	Pre-test score- post-test score	−1.03	0.64	0.12	−1.28	−0.79	−8.71	28	0.00

Table 5 displays the Business Vocabulary pre-test and post-test scores of the experimental group, together with descriptive data. After receiving the treatment, the average Business Vocabulary score of the experimental group climbed from 12.72 on the pre-test to 13.76. The average score on the post-test for Business Vocabulary was 1.04 points higher than the average score on the pre-test.

There is a large performance gap between the Business Vocabulary pre-test and post-test scores, as indicated by the numerical value in Table 6. In addition, the value of the paired sample t-sig test for the pre-test and post-test results of the experimental group is 0.00, which is less than 0.05. The difference between the Business Vocabulary pre-test and post-test scores of the experimental group is 1.03 points, which is statistically significant. The Business Vocabulary post-test scores are much higher than the pre-test scores.

#### 4.1.4. Comparison of business vocabulary pre-test and post-test scores in the control group

**FIGURE 5:**
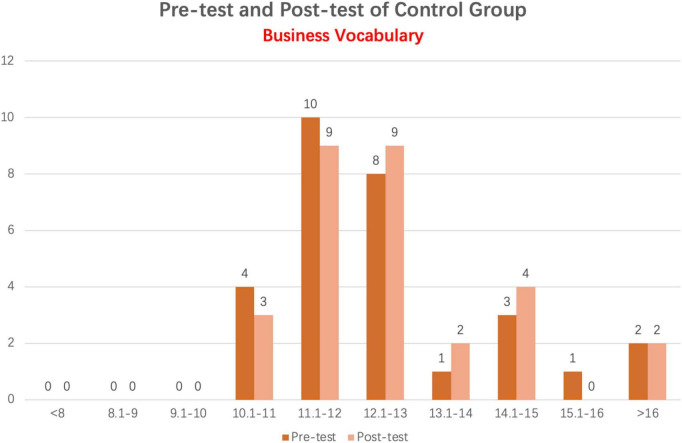
Column distribution of business vocabulary pre-test and post-test scores in control group.

As demonstrated in Figure 5, there is little difference between the pre-test and post-test scores of students in the control group, and the distribution trend in the column figure is also comparable. A majority of students’ scores on the pre-test and post-test were between 11 and 13 points, and the number of students who scored more than 13 points was remarkably similar: 7 on the pre-test and 8 on the post-test.

The difference between the Business Vocabulary pre-test and post-test scores of the control group was analyzed using a paired sample *t*-test. Tables 7, 8 display the respective results.

**TABLE 7:** Paired sample test of business vocabulary pre-test and post-test scores in the control group.

		Mean	*N*	SD	Std. Mean
Pair 1	Pre-test score	12.90	29	1.76	0.33
	Post-test score	12.95	29	1.68	0.31

**TABLE 8:** Paired sample test of business vocabulary pre-test and post-test scores in the control group.

Paired difference									
		**Mean**	**SD**	**Std. Mean**	**95% confidence interval of difference**		***T***	**df**	**Sig.**
					**Lower limits**	**Upper limits**			
Pair 1	Pre-test score- post-test score	−0.05	0.34	0.06	−0.18	−0.08	−0.83	28	0.42

Table 7 presents the descriptive data for the control group’s Business Vocabulary pre-test and post-test results. The average Business Vocabulary pre-test and post-test scores for the control group were 12.90 and 12.95, respectively. The average post-test score for Business Vocabulary is 0.05 points higher than the average pre-test score. Table 8 shows that the numerical difference between the two tests is minor.

From Table 8, the control group’s pre-test and post-test paired sample *t*-test sig value was 0.42, which is greater than 0.05. A significance level of 0.05 was used to demonstrate this. The Business Vocabulary pre-test and post-test results for the control group did not differ significantly.

In response to Research Question 1, findings above revealed that the DingTalk based PBL teaching method can successfully help students in monitoring and using Business vocabulary. On the contrary, it is clear from a review of the results from the control pre- and post-tests that the traditional teaching method is unable to significantly improve students’ ability to use business vocabulary. This demonstrates that the DingTalk based PBL teaching method can improve students’ business vocabulary scores.

### 4.2. Results of research question 2

The responses provided by students to the interview can be used to answer RQ 2. Student A believed that by using DingTalk based PBL teaching method during this period, he was able to use language more precisely business context. Many of his errors had been addressed, and he had gained a greater understanding of the Business context for a large number of words, particularly through group discussion. Student B said that the guidance of the instructor and the class discussion enhanced his capacity to comprehend Business English vocabulary. In addition, the instructor prompted him to apply business vocabulary using DingTalk. Student C stated that after this period of study, he was capable of using business vocabulary more effectively. Student D claimed that timely feedback via DingTalk from the instructor after class have enhanced his use of business vocabulary. In conclusion, DingTalk based PBL teaching method can assist students in enhancing their competence to master and employ Business English vocabulary.

*“I think it can help me use vocabulary more accurately. Especially in the process of group discussion and communication with classmates, it can help me to correct many errors in language expression*…*the meaning of many words in Business scenarios will change, which is also one of the very useful knowledge points I learned during this period.”*

*(SA.19.9.2022.L1-L4).*

“…*through the guidance of teachers and mutual discussion between students in class, I can distinguish Business English words well and use them appropriately, thanks to the careful and timely modification of DingTalk after class.”*

*(SB.19.9.2022.L3-L5).*

“…*I have a better understanding of how to use appropriate language to express problems and solve problems in Business scenarios.”*

*(SC.19.9.2022.L3-L4).*

*“I think there has been a great improvement in the use and selection of vocabulary*…*The teacher’s timely correction on the DingTalk after class also helped me master this method more efficiently.”*

*(SD.19.9.2022.L1-L3).*

### 4.3. Discussion

The discussion serves as the interpretative heart of this study, where the findings obtained from the research are analyzed, synthesized, and contextualized within the broader scope of the study’s objectives. Through a combination of quantitative and qualitative insights, it provides a comprehensive understanding of the impact of DingTalk-based PBL on the business vocabulary growth of Chinese undergraduates during the COVID-19 pandemic. Furthermore, the discussion delves into the implications of the findings and their significance for language education in remote learning contexts.

#### 4.3.1. Impact on business vocabulary growth (RQ1)

The quantitative analysis provided significant insights into the impact of DingTalk-based Problem-Based Learning (PBL) on the growth of business vocabulary among Chinese undergraduates. The results of the pre-test and post-test vocabulary assessments within the experimental group, which received the DingTalk-based PBL intervention, demonstrated a notable improvement in vocabulary scores. Statistical significance was confirmed through paired *t*-tests, indicating that the intervention played a pivotal role in enhancing the business vocabulary of the participants. Comparing the experimental group with the control group, which did not receive the intervention, further solidified the positive effects of the DingTalk-based PBL. This finding resonates with prior research, emphasizing the role of PBL in promoting contextualized language use and active engagement. Notably, these findings align with the works of Elizabeth and Zulida (2012) and Lin (2015). Elizabeth and Zulida (2012) found that PBL not only improved students’ ability to apply grammar and linguistic knowledge but also encouraged the use of more appropriate vocabulary to express content more proficiently.

Similarly, Lin (2015) investigated the impact of PBL on English vocabulary acquisition, demonstrating that students taught using PBL had a superior capacity to apply vocabulary and were more proficient in composing longer texts in English writing. The aforementioned studies collectively emphasize the positive impact of PBL on students’ vocabulary use in the English language. Our study, however, extends this scope into the realm of business English and explores whether PBL can facilitate a deeper understanding of the use of business English vocabulary. Chen et al. (2021) investigated how the combination of virtual reality technology and a PBL setting influenced students’ motivation during the English learning process and their vocabulary acquisition. Their findings, unlike those mentioned earlier, demonstrated the effectiveness of the PBL + DingTalk teaching method in helping students master more business vocabulary, select appropriate business words, and apply them effectively in a business context.

In summary, our study supports the existing body of literature by affirming the positive influence of PBL on language acquisition, particularly in a business context. Furthermore, it introduces a novel approach, combining PBL with modern educational technology, to enhance students’ business vocabulary learning. This approach, as evidenced by our results, offers a promising method for students to develop their language skills and practical vocabulary in the context of business English.

#### 4.3.2. Attitudes toward DingTalk-based PBL (RQ2)

Qualitative insights from semi-structured interviews shed light on students’ attitudes toward DingTalk-based PBL during the COVID-19 pandemic. Participants from experimental group expressed generally positive attitudes, citing benefits such as increased motivation, improved interaction with course content, and enhanced collaboration with peers. The platform’s incorporation of authentic scenarios and multimedia resources contributed to a dynamic and engaging learning experience. While challenges such as technical issues and adjusting to virtual collaboration were mentioned, participants’ overall sentiments highlighted the potential of DingTalk-based PBL to bridge the gap between traditional classroom settings and remote learning.

The responses of the students to the interview questions can be utilized to research how students feel about the effects of DingTalk based DingTalk teaching method in terms of business vocabulary learning. Student A highlighted the collaborative aspect of the approach, which enabled them to acquire a diverse set of business vocabulary through communication and teamwork when addressing real-world business challenges. This collaborative problem-solving aspect of PBL is a key component in the virtual learning design. Student B emphasized the value of timely feedback provided by instructors through DingTalk, contributing to the enhancement of language skills and the effective use of business vocabulary. The timely feedback element is another integral aspect of PBL that was employed in the virtual learning context. Students C and D echoed the sentiments of Student A and Student B, underscoring the positive impact of DingTalk-based PBL on their vocabulary acquisition and expression. They also highlighted how the platform facilitated effective communication with both instructors and fellow students, which aligns with the collaborative nature of PBL.

These responses collectively underscore the effectiveness of DingTalk-based PBL in enhancing business vocabulary learning. Through in-class discussions, collaborative problem-solving, and post-class feedback, students were able to use business vocabulary with greater precision and achieve higher quality outcomes in Business English tasks across diverse language requirements in various business contexts. By drawing these explicit connections between the observed outcomes and the components of the PBL technique utilized in our virtual learning design, we provide a more in-depth understanding of how DingTalk-based PBL facilitated vocabulary acquisition and improved students’ attitudes toward language learning.

#### 4.3.3. Implications for language education

The implications drawn from the findings of this study hold significant value for the field of language education, particularly within the evolving landscape of remote learning, such as that necessitated by the COVID-19 pandemic. The integration of DingTalk-based problem-based learning (PBL) has several far-reaching implications that extend beyond the scope of this study.

##### 4.3.3.1. Enhancing remote learning engagement

The positive attitudes expressed by participants toward DingTalk-based PBL underscore its potential to mitigate the detachment often associated with remote learning environments. By incorporating authentic scenarios, multimedia resources, and collaborative activities, DingTalk’s platform provides learners with dynamic and engaging learning experiences. These findings suggest that such innovative approaches can reinvigorate learners’ engagement and motivation, which are crucial factors for effective language acquisition in virtual settings.

##### 4.3.3.2. Fostering collaborative language learning

The collaborative nature of PBL facilitated by DingTalk encourages peer interaction and shared problem-solving. This collaborative learning environment not only simulates real-world interactions but also aligns with the constructivist principles of language acquisition. Language proficiency thrives when learners engage in meaningful conversations, negotiate meaning, and collaborate to address linguistic challenges. As such, DingTalk-based PBL’s emphasis on collaboration can be adapted across various language education contexts.

##### 4.3.3.3. Bridging physical and virtual learning spaces

The COVID-19 pandemic underscored the need for innovative solutions that bridge the gap between physical classrooms and virtual learning spaces. DingTalk-based PBL offers a seamless transition by recreating the collaborative, problem-solving aspects of classroom interactions in an online format. This suggests that even in post-pandemic times, such platforms can continue to serve as valuable tools that offer continuity in language education, catering to both in-person and remote learning scenarios.

##### 4.3.3.4. Reimagining language assessment

The significant improvement in business vocabulary scores observed among participants who underwent DingTalk-based PBL indicates the potential for reshaping language assessment methods. Traditional summative assessments might not fully capture the depth of language learning facilitated by problem-based learning. This study suggests that incorporating more contextually relevant and dynamic assessments, such as scenario-based tasks, could better reflect learners’ practical language skills.

##### 4.3.3.5. Pioneering technology-enhanced language pedagogy

DingTalk’s platform showcases the integration of technology and pedagogy, presenting educators with opportunities to pioneer new instructional methods. The interactive nature of the platform, coupled with its authentic scenario design, exemplifies how technology can enhance language instruction beyond the confines of the traditional classroom. This study encourages educators to explore and harness the potential of similar platforms to cultivate immersive language learning experiences.

#### 4.3.4. Limitations and future research

While this study provides valuable insights into the effects of DingTalk-based problem-based learning (PBL) on Chinese undergraduates’ business vocabulary growth during the COVID-19 pandemic, it is essential to acknowledge its limitations and consider avenues for future research to expand upon the findings.

##### 4.3.4.1. Short-term focus

One of the limitations of this study is its emphasis on short-term outcomes. The pre-test and post-test vocabulary assessments provided insights into immediate vocabulary growth following the intervention. However, language acquisition is a dynamic process that involves both short-term and long-term retention. Future research could explore the sustainability of vocabulary growth over an extended period, shedding light on the effectiveness of DingTalk-based PBL in fostering enduring language proficiency.

##### 4.3.4.2. Context-specific findings

This study was conducted within a specific context of Chinese undergraduates during the COVID-19 pandemic. The results may be influenced by the unique dynamics of this context, including technological familiarity and cultural factors. To enhance the generalizability of the findings, future research could replicate the study in diverse language learning environments, involving participants from different cultural backgrounds and educational levels.

##### 4.3.4.3. Exploring multidimensional language skills

While this study focused on business vocabulary growth, language acquisition encompasses various dimensions, including speaking, listening, reading, and writing. Future research could broaden its scope to investigate the impact of DingTalk-based PBL on other language skills. This expansion would provide a more holistic understanding of how the platform affects learners’ overall language proficiency and communication abilities.

##### 4.3.4.4. Long-term attitudinal changes

The qualitative insights captured participants’ attitudes toward DingTalk-based PBL during the intervention period. Exploring how these attitudes evolve over time and persist in subsequent language learning experiences could offer insights into the long-term impact of the platform on learners’ perceptions and motivations. Longitudinal studies could shed light on how DingTalk-based PBL influences learners’ broader language learning journeys.

##### 4.3.4.5. Hybrid learning models

As education continues to evolve toward hybrid learning models that combine in-person and virtual elements, research could investigate how DingTalk-based PBL can be integrated within such hybrid contexts. Exploring how the platform complements traditional classroom settings while maintaining the benefits of remote learning could provide valuable insights for educators navigating post-pandemic educational landscapes.

## 5. Conclusion

This research embarked on a journey to explore the transformative potential of DingTalk-based problem-based learning (PBL) on Chinese undergraduates’ business vocabulary growth during the COVID-19 pandemic. The findings of this research underscore the efficacy of DingTalk-based PBL in enhancing Chinese undergraduates’ business vocabulary. The implications of this research reach beyond the confines of this study. DingTalk-based PBL has the potential to redefine language education, particularly in the context of remote learning and crises like the COVID-19 pandemic. It offers innovative solutions to enhance engagement, cultivate collaboration, and facilitate meaningful language acquisition. These implications extend to the domains of technology-enhanced pedagogy, assessment methodologies, and the ongoing evolution of hybrid learning models. As educators, learners, and researchers, we stand at the crossroads of innovative pedagogy and technology-driven learning experiences, armed with insights to drive transformative change in language education.

By examining DingTalk-based PBL’s impact on vocabulary growth and attitudes, this study offers a foundational understanding that invites further investigation and application. As the educational landscape continues to evolve, the insights gleaned from this research contribute to the ongoing dialog on how technology can enrich language learning experiences. This research journey concludes with a vision of a future fuelled by innovation, where platforms like DingTalk catalyze not only language acquisition but also a broader paradigm shift in education. As educators and learners alike harness the power of technology-mediated pedagogy, the journey toward dynamic, effective, and engaging language education continues to evolve, leaving an indelible mark on the trajectory of learning in the digital age.

## Data availability statement

The original contributions presented in the study are included in the article/supplementary material, further inquiries can be directed to the corresponding author.

## Ethics statement

The studies involving humans were approved by the Harbin Institute of Petroleum Ethics Committee. The studies were conducted in accordance with the local legislation and institutional requirements. The participants provided their written informed consent to participate in this study.

## Author contributions

LS: Conceptualization, Formal analysis, Investigation, Methodology, Writing – original draft. HD: Data curation, Investigation, Supervision, Writing – review and editing. XZ: Formal analysis, Validation, Writing – review and editing.
